# Supplementary material for: Impact of Nanoclay on the pH-Responsiveness and Biodegradable Behavior of Biopolymer-Based Nanocomposite Hydrogels
Source: Gels. 2019 Oct 16;5(4):44. doi: 10.3390/gels5040044 (PMC6955902; doi:10.3390/gels5040044)
Supplement: Supplementary file 1 [file gels-05-00044-s001.pdf]

Supporting Information

Impact of Nanoclay on the pH-Responsiveness and Biodegradable Behavior of Biopolymer-Based Nanocomposite Hydrogels

Arti Vashist [1,2,] Anujit Ghosal [1,3], Atul Vashist [4], Ajeet Kaushik [2,5], Y.K. Gupta [6], Madhavan Nair [2] and Sharif Ahmad [1,*]

*Corresponding author: Tel: +91 9891290510, Email: sharifahmad_jmi@yahoo.co.in

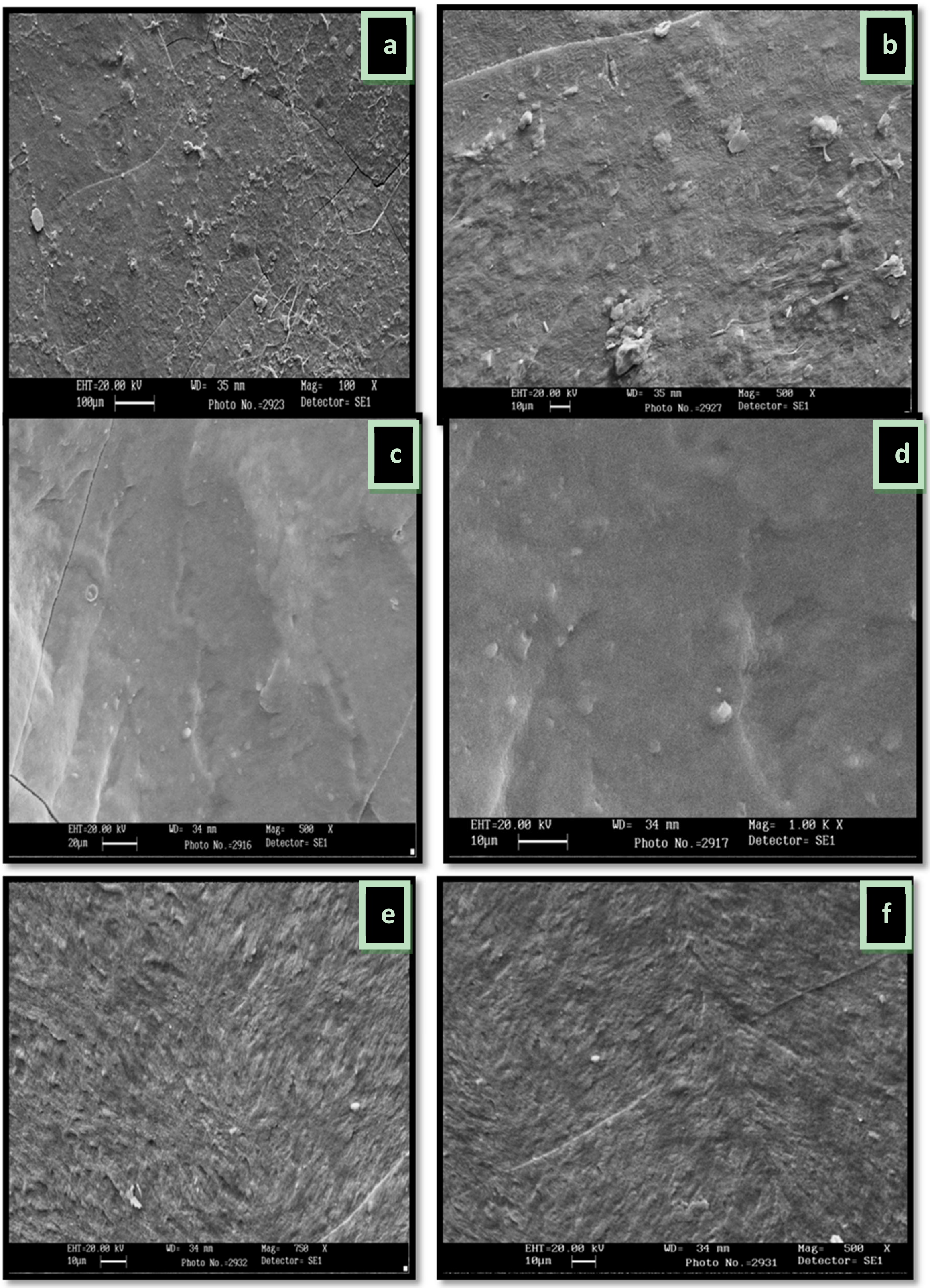

Figure S1. SEM micrographs of CG-0 (**a** and **b**), CGP2-3 (**c** and **d**) and CGP2-5, (**e** and **f**) dried hydrogels.

TGA analysis:

A 10 wt% loss occurred within the range of temperature 150°C - 200 °C, due to loss of imbibed solvent molecules from the hydrogels. The 50 wt% loss occurred in the temperature range of 370 °C to 400 °C. This may be attributed to the decomposition of ether and amide linkages. The third weight loss occurred in the temperature range 450 °C -480 °C and beyond this point, the degradation continues up to 700 °C. The enhanced thermal stability of hydrogels CGP2-1 to CGP2-5, as imparted by polyol induced cross linking is evident by the increase in degradation temperature of these hydrogels. The enhanced thermal stability of CGP2-5 in comparison to other ratios can be attributed to the formation of highly cross linked network containing more amount of polyol, which act as a cross linking agent, thus, enhancing the stability of the hydrogel network.

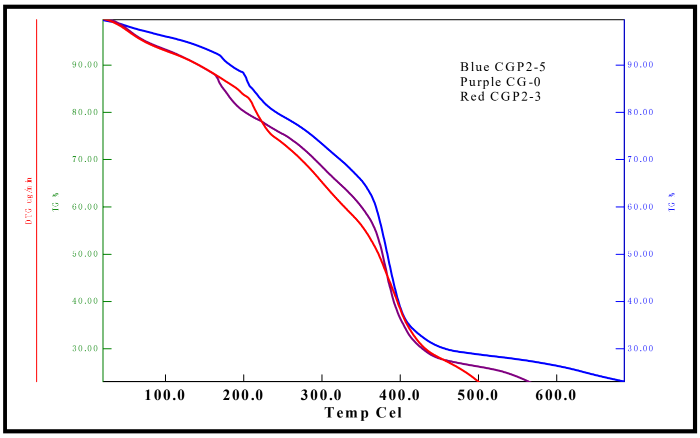

Figure S2. TGA analysis of CGP2-5, CG-0 and CGP2-3 hydrogels.

DSC analysis: The glass transition temperature was observed in the temperature range 45-50° with enthalpy relaxation.[37] The composition CGP2-3 and CGP2-5 exhibits exothermic peaks around 225-250ºC as well as at around 400ºC. A shift in exothermic peaks to higher temperatures was observed for CGP2-5 and CGP2-7 prepared with a higher concentration of polyol. These results indicate that the energy needed for the fusion of polymer increased with an increase in cross-linking, and support the fact that an increase in cross-linking increases the polymer chain rigidity and hence, higher energy is required to break the highly cross-linked polymer than the loose network.

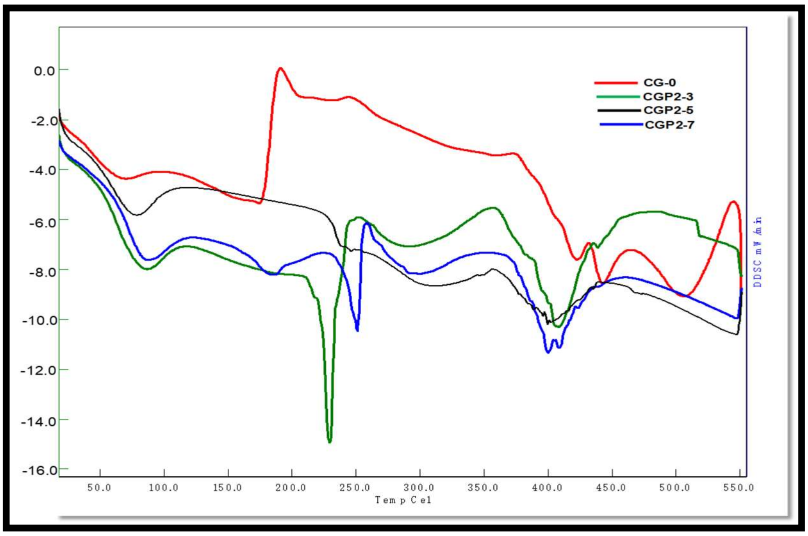

Figure S3. DSC micrographs of CH-guargum-polyol hydrogels.

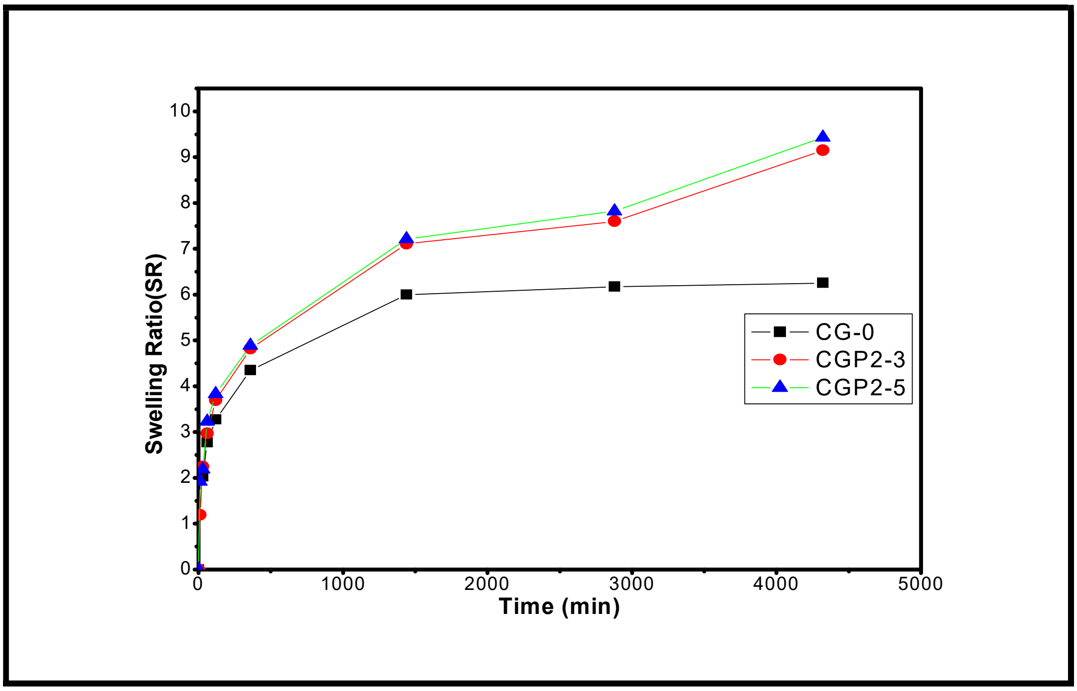

Figure S4. Swelling ratio of CG-0, CGP2-3 and CGP2-5 at pH 7.4.

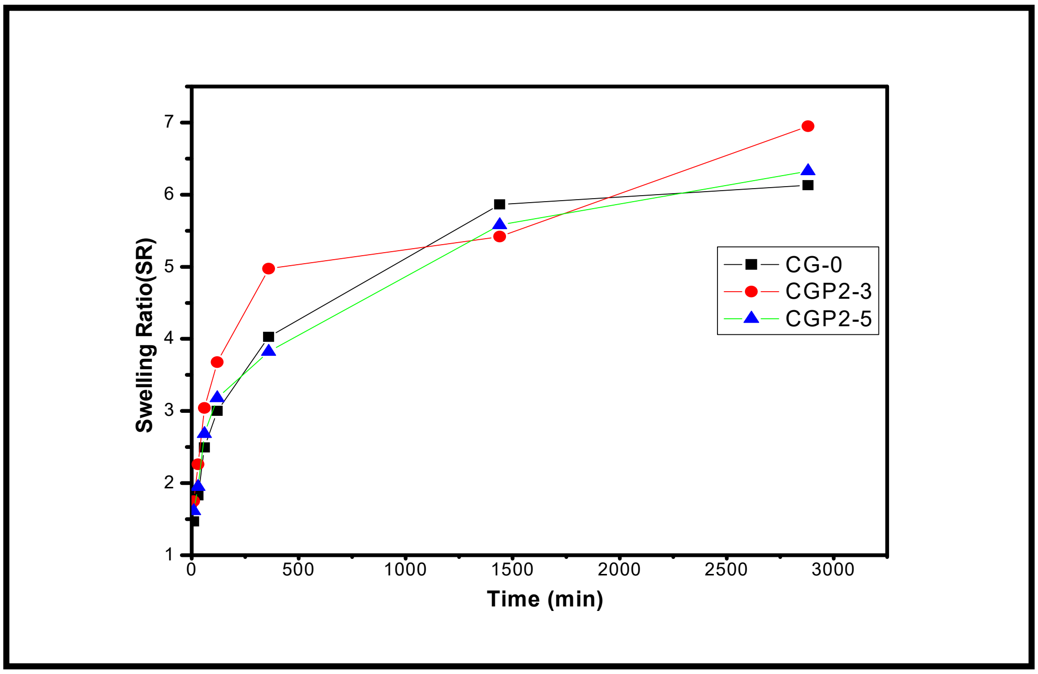

Figure S5. Swelling ratio of CG-0, CGP2-3 and CGP2-5 at pH 4.

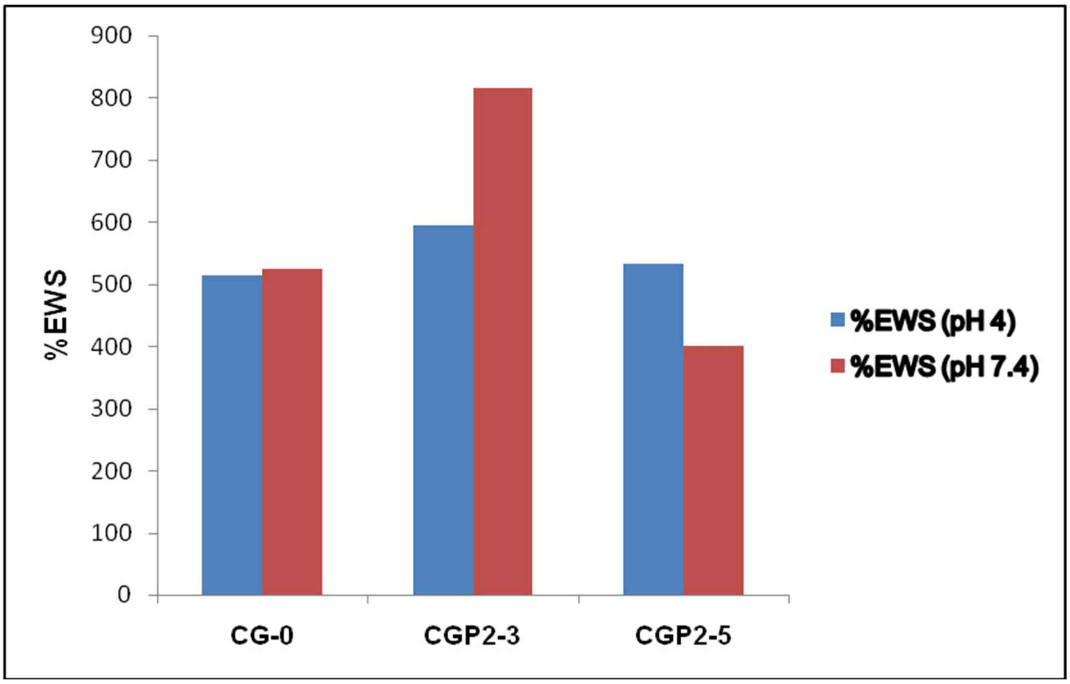

Figure S6. % EWS and pH relationship in pH 4 and pH 7.4 buffer solutions.

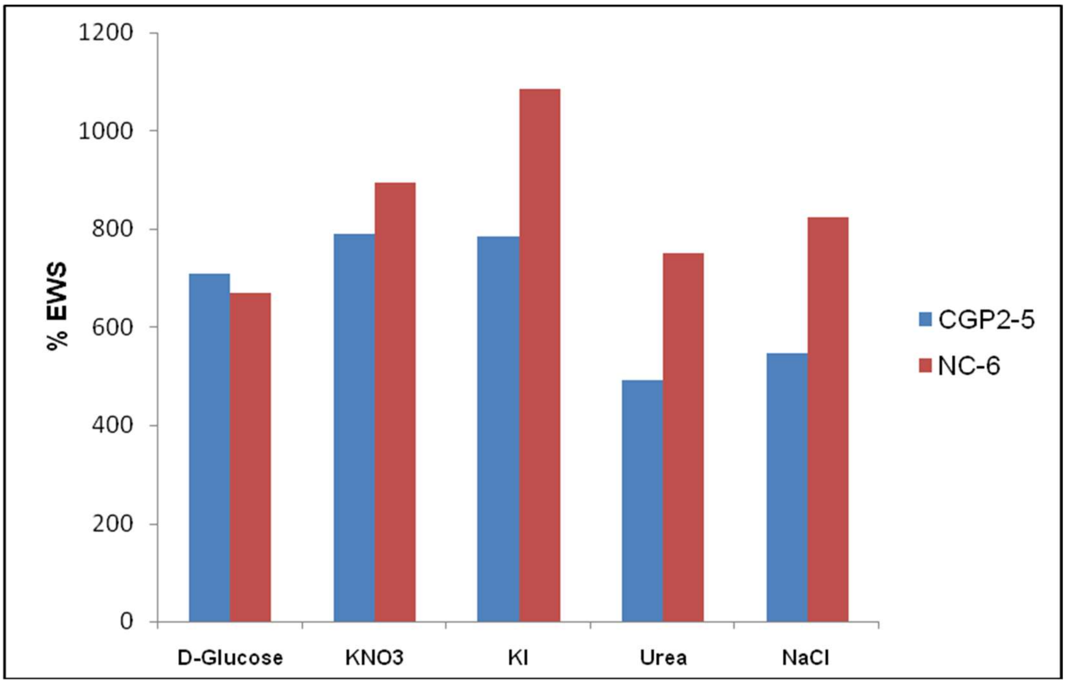

Figure S7. Effect of physiological solutions on %EWS of CGP2-5 and NC-6 hydrogels.

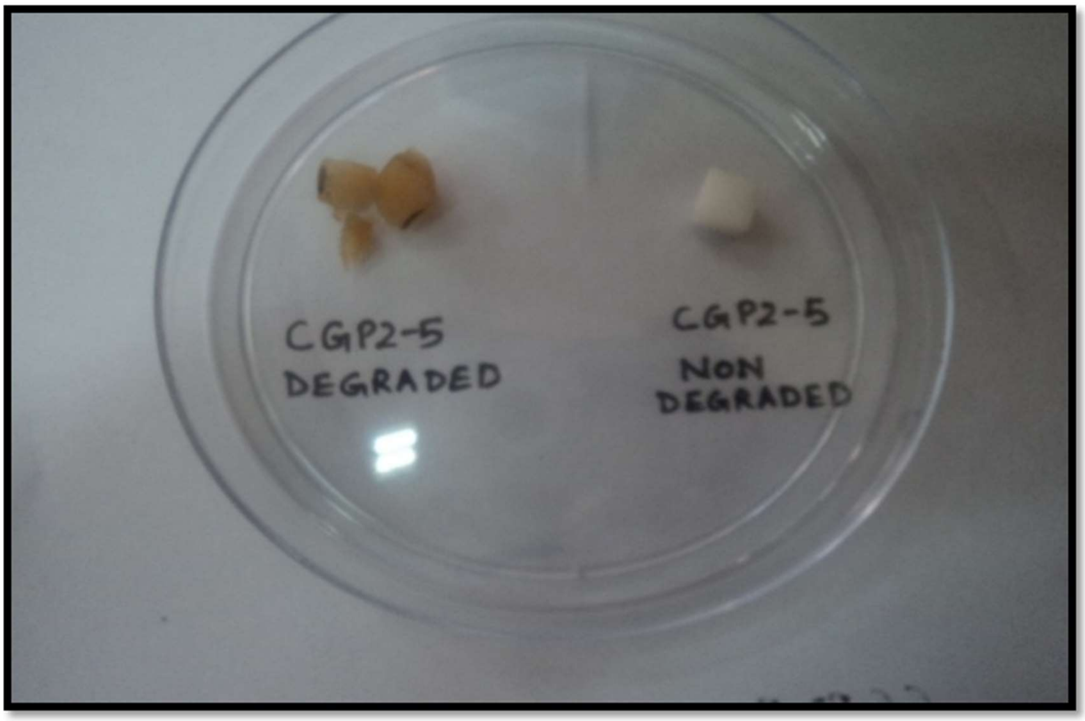

Figure S8. Digital picture of CGP2-5 hydrogel before and after the soil burial degradation study.
